# Stroke incidence in the young: evidence from a Norwegian register study

**DOI:** 10.1007/s00415-018-9102-6

**Published:** 2018-10-30

**Authors:** Mathias Barra, Angela S. Labberton, Kashif W. Faiz, Jonas C. Lindstrøm, Ole Morten Rønning, Joe Viana, Fredrik A. Dahl, Kim Rand

**Affiliations:** 1 The Health Services Research Unit-HØKH, Akershus University Hospital, Lørenskog, Norway; 2 Centre for Connected Care, Oslo University Hospital, Kirkeveien 166, Oslo, Norway; 3 Institute of Clinical Medicine, Faculty of Medicine, University of Oslo, Oslo, Norway; 4 Division of Medicine, Akershus University Hospital, Lørenskog, Norway

**Keywords:** Stroke incidence, Epidemiology, Case fatality, Regression modelling, Cerebrovascular accident

## Abstract

**Introduction:**

While there is a general agreement that stroke incidence among the elderly is declining in the developed world, there is a concern that it may be increasing among the young. The present study investigates this issue for the Norwegian population for the years 2010–2015. Cerebrovascular accidents (CVAs) for patients younger than 55 years were identified through the Norwegian Patient Registry and the Norwegian Cause-of-death Registry.

**Methods:**

Negative binomial regression modelling was used to estimate temporal trends in the CVA incidence rates for the young, aged 15–54, with 10-year sub-intervals, and for children below the age of 18. The main outcomes were CVA incidence per 100,000 person-years at risk (PY), 30-day stroke mortality per 100,000 PY, and 30-day case-fatality rates.

**Results:**

The analysis showed a negative and non-significant temporal trend in the CVA incidence ($p = 0.052$) as well as for 30-day mortality ($p = 0.074$) for the age group 15–54. Overall, the inclusion of an interaction for age in the bracket 45–54 suggested that any temporal decline is restricted to this age bracket. The analyses of the 10-year age brackets 15–24, 25–34, and 34–45, provided evidence neither for an increase, nor for a decrease, in incidence. Among the children, the estimated temporal coefficients were positive, but non-significant, consistent with a stationary trend.

**Conclusion:**

Weak statistical evidence was found for a decline in CVA incidence and for overall stroke 30-day case fatality for 15–54 year olds, but the decline was significant only for the 45–54 age band. All results considered, the study suggests a stationary or decreasing temporal trend in CVA incidence and stroke fatality for children (0–18) and young (15–54) in Norway. Even larger data sets are needed to estimate these temporal trends accurately.

## Introduction

Recent studies have suggested that the incidence of stroke, one of the main contributors to premature death and disability, has declining in the developed world [1–5]. However, there is concern that this overall trend masks an alarming increase in incidence among the young—often defined as those below the age of 55 [6]. Because stroke incidence has a very strong age gradient, with an exponential growth curve as the individual ages, most strokes originate from the older cohorts. Hence, even a moderate decrease in age-adjusted stroke incidence for the elderly could potentially disguise a significant increase in stroke incidence among the younger segments of the population. As stroke etiology differs between the old and the young, [7] the successful efforts in recent decades to better detect and manage stroke risk factors may not be as effective in the younger segments of the population as in the elderly.

The influential Journal of the American Heart Association recently printed an editorial titled *Rising Stroke Incidence in Young Adults: More Epidemiological Evidence, More Questions to Be Answered* by Y. Béjot et al. with a call for more research into this phenomenon [8]. This editorial states that ‘[t]here is now a large amount of evidence that ischemic stroke incidence is on the rise in young adults, and the reasons for this trend are probably multiple’[8]. Several possible reasons for a rise in stroke incidence among the younger cohorts in the developed countries are suggested. An increase in classical vascular risk factors, alongside the emergence of new risk factors such as substance abuse, is discussed alongside possible confounders such as an increased awareness and better stroke recognition.

Articles cited in the editorial as evidence for increasing stroke in the young are listed in Table 1, as well as additional relevant studies. Many of the articles cited as supporting an increasing incidence offer alternative explanations for the observed increase, and are substantially less categorical in their conclusions. For example, Kissella et al. highlight that, while their study demonstrated an apparent increase in stroke incidence (including subarachnoid haemorrhage) in the young, they also documented that the fraction of patients for whom an MRI was obtained roughly doubled during the study period (from 37.5 to 70.0% for 20–44 years old, and from 31.0 to 63.4% for 45–54 years old.) Hence, a temporal trend in the health systems’ ability to correctly detect strokes in the younger segments of the population could be a possible confounder.

**Table 1:** Selected stroke in the young literature

Author	Publ. year	Location	Diagnoses	Period	Age brackets	Incid.	Mort.
Medin [9]	2004	Sweden	I61, I63	1989–1991 vs. 1998–2000	30–49, 50–59, 60–65	+	−
Mallick [10]	2010	UK	I61, I63	1921–2000	0–18	NA	+ then −
George [11]	2011	USA	I61, I63	1995–2008	5–14, 15–34, 35–44	+	0
Kissela [12]	2012	USA	I60, I61, I63, I64	1993–1994 vs. 1999 vs. 2005	20–54	+	NA
Gonzaáles-Pérez [13]	2013	UK	I60, I61	2000–2008	20–49, 50–59	NA	−
Rosengren [14]	2013	Sweden	I63	1987–2010	18–44, 45–54, 55–64	+	−
Vaartjes [15]	2013	Netherlands	I63	1997–2005	35–64	+	−
Béjot [6]	2014	Netherlands	I63	1993–2007	$<55$	+	NA
Poisson [16]	2014	USA	I63	1989–2009	20–45	+	−
Krishnamurthi [17]	2015	World (GBD)	I61, I63	1990 vs. 2013	0–18	−	−
Ramirez [18]	2016	USA	I63	2000–2010	25–44, 45–64	+	NA
Tibæk [19]	2016	Denmark	G45, I61, I63, I64	1997–2006 vs. 2007–2012	15–30	+	NA

*Incid./Mort.* finding w.r.t. incidence/mortality (+ = increasing; − = decreasing; 0 = stationary; *NA* not applicable). *GBD* global burden of disease; *G45* transient ischemic attack (TIA), *I60* subarachnoid hemorrhage, *I61* Intracerebral haemorrhage, *I63* ischemic stroke, *I64* undetermined stroke. Rosengren tested 1987–1992 vs. 1993–1998 vs. 1999–2004 vs. 2005–2010

Another important point in this context is that several of the studies indicating an increase in stroke incidence, only consider hospitalised strokes, which does not imply that stroke incidence is increasing. In particular, if the severity of hospitalised strokes show a trend towards milder strokes, what we could be observing is not an increase in stroke incidence, but rather an increase in the health systems’ ability to detect, diagnose, and treat more of the milder strokes. Assuming an underlying constant stroke incidence, we would expect an increase in the number of diagnosed stroke events given the remarkable development in imaging techniques, and their increased use in clinical practice.

It is also problematic that the category young adults is not uniformly defined, and that methods and classification vary greatly between studies, both with respect to incidence rates and aetiology [20]. Hence, it is hard to judge the evidence presented in the articles in Table 1 collectively. The individual risk of stroke increases exponentially with age [21–23]. Estimated incidence rates can therefore be quite sensitive to choice of age strata during analyses. For example, if 20–44 years old are grouped together in a regression model, and one aim is to estimate the association between year and stroke incidence, even a moderate shift of the age-stratified composition of the 20–44 age group—i.e., towards ‘more 44 years old and less 20 years old’—could be confounded with a temporal trend towards increased cerebrovascular accidents (CVA) incidence, due to the underlying exponential increase in individual risk for stroke or transient ischemic attack (TIA).

Some contemporary articles point to evidence of either a stationary, or even declining, trend [24, 25]. Notably, to our knowledge, no studies have demonstrated increased stroke-fatality rates in the young. This is true both in the sense that there has been no increase in fatalities attributable to strokes, nor has there been an increase in the case fatality rates—the proportion of registered strokes resulting in death—quite the contrary [15].

While the recent shift in acute stroke treatment—from life-supporting and palliative care to effective and advanced interventions—has been a great success in terms of better outcomes for survivors, very few studies have shown improved survival. Therefore, if stroke incidence is on the rise—within any sub-population—we would expect an accompanying rise in fatalities. In the case of the young, the observed combination of increased incidence and declining mortality could be fully explained by increased sensitivity to milder strokes, whereas assuming an underlying increase in stroke incidence would need to be substantiated with a separate rationale for the lack of corresponding increase in fatality.

Vaartjes et al. found that while stroke incidence appears to be increasing in the Netherlands, stroke fatalities are declining—both in absolute terms (i.e., deaths following a stroke per 100k)—and as a percentage of those with a stroke [15]. The authors note that the second statistic shows the most dramatic decline, and acknowledge that they do not have case mix data: i.e., their finding is consistent with an increase in minor, less severe strokes only. They also remark that TIAs are increasing more than other diagnoses during 1995–2010, and tentatively conclude that minor strokes may be the reason behind the observed overall stroke increase. However, no consideration is given to the possibility that the observed increase may be due to an increased propensity for hospitalising less severe strokes, and not that there are necessarily more of them. For example, in a recent Norwegian study, it was shown that during the six months following a mass media intervention aimed at increasing public awareness of stroke symptoms, the average number of ED arrivals on indication suspected stroke almost doubled [26].

Due to uncertainties regarding current changes in stroke incidence in the young, the aim of this study is to investigate temporal trends in childhood and young stroke incidence and fatality, during recent years, using a large data set of all CVAs obtained from the Norwegian Patient Registry (NPR) and the Cause-of-death Registry (NCDR) in Norway for 6 years, 2010–2015 (received May 2017).

This study provides another piece of the puzzle of recent temporal trends in CVA incidence in children and the young, broken down by TIA, ischemic, haemorrhagic, and undetermined stroke. In this way, we provide a more complete picture than is commonly provided in the literature, and also hope to set a standard for the reporting on CVA sub-types together, so that possible shifts between e.g., TIA and ischemic stroke are not confounded with an increase or decrease. On this note, this is also the first study to consider temporal trends in TIA incidence in the young on a relatively large data set; the only former large study with a separate focus on TIA that we know of considered all ages [27].

## Methods

The data used in this study is described in greater detail elsewhere [28]. Briefly, we combined data from two sources: the NPR, and the NCDR. NPR collates hospital records, including demographics, ICD-10 codes, and admission times from all hospitals in Norway that treat CVA. NCDR collates corresponding information regarding all deaths in Norway. Our data set therefore contains hospital and fatality records for all persons recorded with at least one stroke diagnosis (ICD-10 codes I61, I63, I64) or TIA (ICD-10 code G45) in either NPR or NCDR between 2010 and 2015; the longest time period for which data from both registers were available and could be linked. This dataset was combined with Statistics Norway records for the total Norwegian population, broken down by year, 1-year age groups, and sex. Recorded stroke episodes for the same individual^1^ were merged when on adjacent dates. This was done separately by sub-diagnosis (ischemic, haemorrhagic, undetermined and TIA), and jointly for strokes. Subsequent to merging, episodes were assigned to year by their start date. As such the data set does contains all events of hospitalised stroke, in addition to all events where a stroke lead to a fatality. We have little reason to believe that there are many non-fatal strokes that do not lead to hospitalisation in particular not among children or the young. Since TIAs do not lead to deaths by definition, our counts of TIAs is for hospitalised TIAs. However, the threshold for admitting TIAs are low (a recent study showed up to 40% of admissions to a large stroke unit in Norway were stroke mimics [29]) and mandated by national guidelines. This means that we believe our counts are fairly complete, and that only a negligible share of the CVAs experienced by the 0–54 cohorts will not be represented in our data set.

The following ICD-10 diagnosis codes were used for case ascertainment: I63 (ischemic stroke), I61 (haemorrhagic stroke), I64 (undetermined stroke) and G45 (TIA).^2^ Strokes denote the aggregated category ischemic, haemorrhagic, and undetermined, but when computing the counts, events that occurred within 7 days of each other was collapsed onto one stroke event to avoid double counting. Cerebrovascular accidents (CVAs) denotes strokes and TIAs combined. We believe it is prudent to perform all analyses for all of these categories where the data permits; in particular when researching the question about possible temporal trends, since diagnostics, stroke awareness, and hospitals may vary in their practice and ability to distinguish between these categories.

The main outcome of interest was CVA incidence per 100,000 person year at risk (PY), pooled over all CVAs and for the specific CVA sub-types. Furthermore, we analysed mortality trends by two statistics: stroke mortality—here defined as total number of 30-day case fatalities per 100,000 PY, and, case fatality rates. The former is the number of recorded deaths within 30 days of a recorded stroke (including non-hospitalised cases). The case fatality rate is the fraction of the strokes that led to death within 30 days, and as such is relative to the incidence statistic. All three outcomes convey different information, are sensitive to different confounders (actual CVA incidence, case mix, health systems sensitivity for CVA and their ability to distinguish them, treatment options), and may exhibit different temporal trends.

In this study, we are investigating temporal trends in the age and sex-adjusted CVA incidence rates in the young. To answer this question, several concerns must be addressed: first, the definition of ‘the young’. We have considered both childhood stroke—defined as stroke in individuals aged 0–18—and stroke in the young—defined as individuals aged 15–54. This delineation was chosen because it corresponds well with most of the classifications from the literature on stroke in the young.^3^

Since we are modelling count data it is reasonable to consider the negative binomial distribution; i.e., assuming that $C_{y,a,s}\sim \mathrm {NB}(\mu _{y,a,s},\theta )$, where $C_{y,a,s}$ is the number of CVA events of interest for year *y*, age *a*, and sex *s*. This leads to the model specification $\mu _{y,a,s}=\text {PY}_{y,a,s} e^{\beta _0+\beta \mathrm {Year}+ \beta _a\mathrm {Age}+ \beta _s\mathrm {Sex}}$. That is, the expected number of events is modelled as a function of year, age, and sex, with a factor PY to account for the population size in the bracket. The residuals distributed as a negative binomial with dispersion parameter $\theta$. The negative binomial model is parameterised so that the variance is $\mu + \mu ^2/\theta$ and converges to the Poisson distribution as $\theta$ become large. The main coefficient of interest here is thus the $\beta$ on the year variable, which should be able to capture temporal trends.

These were fitted both with the counts of stroke events and 30-day stroke fatalities as the dependent variable. This was done for childhood stroke (0–18), and for stroke in the young (15–54), and furthermore, separate models were fitted for each 10-year sub-group 15–24, 25–34, 35–44, 45–54. We fitted models for each of the stroke categories I61, I63, I64, G45, and for the aggregate categories strokes and CVAs. Furthermore, we specified separate models for men and women [30].

We also analysed overall 30-day case fatalities, because this outcome should be sensitive to an increase in underlying incidence rate. Strokes are life-threatening, and under the assumption that the number of stroke fatalities is more readily available than the number of stroke events, it seems reasonable to expect that an increase in stroke incidence would be accompanied by an increase in stroke fatalities. As sensitivity analyses, we also performed Mann–Kendall non-parametric test for temporal trends, [31, 32] and compared the incidence rates in 2010–2011 vs. 2014–2015 by a standard Chi square test for difference in proportion over the same groups. We also computed the relative share of haemorrhagic, ischemic, undetermined, and TIA of all CVAs, stratified by age group and inspecting for temporal trends.

All analyses were performed with the statistical software R in the RStudio environment, [33, 34] relying on the Plotly and Stargazer packages for graphing and rendering regression outputs [35, 36]. The negative binomial regression model was fitted with the MASS-package’s glm.nb-function [37].

### Ethics

The study was conducted as part of a larger project aimed at modelling patient flow for stroke patients in the Norwegian healthcare system. The project has been considered and approved by the Regional Committees for Medical and Health Research Ethics (reference 2015/1009). The national registries providing data are obliged by law to conduct their own judgement of research projects prior to granting access to data. Access to data was approved by the National Patient Registry and the Norwegian Cause of Death Registry. Sensitive data were handled in accordance with regulations and procedures required by law and implemented by Akershus University Hospital; in particular the terms of release from NPR and NCDR prohibit depositing the raw data to a public data repository.

## Results

### Descriptive Statistics

The full NPR data set contained data on a total of 105,792 CVAs, accounting for 30,090,507 PYs, and resulting in a total of 17,915 fatalities for the 6 years 2010–2015. The childhood group (0–18) covered 7.1M (23.6%) PYs and 270 (0.3%) CVAs, and nine fatalities; 51.3% were male, and mean age was 9.1 years. The young (15–54) accounted for 16.3M (54.2%) PYs, 8 973 CVAs (8.5%), and 279 (1.6%) fatalities in total; 51.3% were males and mean age was 34.8 years. The mean ages of the sub-groups were all at within 0.3 years of the centres of the intervals. More detailed, aggregated counts, broken down by CVA, age, and sex categories, are presented in Table 2. This table also displays crude incidence, fatality, and case fatality rates; separated by CVA (and, for the young, sex) category, for childhood and young stroke. Take note that the stroke category is not equal to the sum of ischemic, haemorrhagic and undetermined strokes, since two events close in time are merged. CVA is the sum TIA + strokes.

The population at risk is relatively healthy, wealthy and stable: in Norway, there are about 30,000 males and females born each year over last 60 years, with a life expectancy at birth rising from 73.5 (1960) to 82 (2015) years; child mortality is in general low, GDP per capita is high, and health care is free and universal. Statistics Norway has high-quality data on Norwegian demographics, which are also available in English language pages.^4^

### Childhood stroke ($\mathrm {age}\le 18$)

**Fig. 1:**
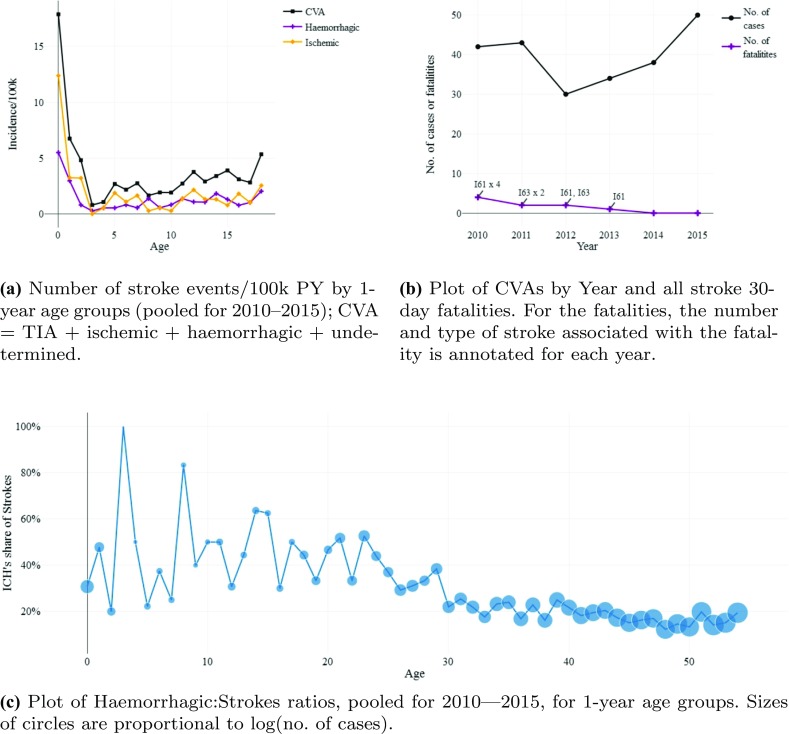
Graphical depiction of: **a** childhood CVA incidence by age; **b** number of cases and fatalities by year; and (1c) the fraction of strokes that are haemorrhagic (ICH—includes stroke in the young)

Despite having access to all CVAs in children, the data set is rather sparse due to a low number of CVA events, with only 270 registered CVAs, and 9 fatalities, in total. However, the pooled data set clearly demonstrates a ‘hockey-stick’ shape for childhood CVA incidence: a sharp decline from birth towards the 3 years of age followed by a moderate increase thereafter; see Table 2 and Fig. 1a.

Due to very few recorded TIAs (33 in the whole period) and undetermined strokes (six events), regression models were only estimated for the ischemic, haemorrhagic, and strokes categories. Furthermore, due to the non-monotonous age profile on stroke incidence noted above, the regression models for childhood strokes included a dummy $\mathrm {Age}_{\le 3}$ coded as 1 for for age below four years, as well as its interaction with age, yielding the specification

$$C_{y,a,s}=\beta _0+ \beta \mathrm {Year}+ \beta _a\mathrm {Age} + \beta _{\le 3}\mathrm {Age}_{\le 3} + \beta _{a\times \le 3}\mathrm {Age}\times \mathrm {Age}_{\le 3}+\beta _s\mathrm {Sex}.$$

#### Incidence: CVAs per 100,000 PYs

Nine models for childhood stroke incidences were estimated. For each of the three diagnosis groupings, the following models were estimated (Table 3): 1. without $\mathrm {Age}_{\le 3}$-dummy; 2. with $\mathrm {Age}_{\le 3}$-dummy; 3. with non-significant variables omitted.

Year was not a significant predictor for childhood stroke, i.e., there was no evidence for a temporal trend, but the coefficients were positive.

The coefficient on age is negative in the models without $\mathrm {Age}_{\le 3}$-dummy interaction, reflecting the very steep decline in stroke incidence between birth and age three (see also Fig. 1a). However, when the $\mathrm {Age}_{\le 3}$-dummy is included, the coefficient on age become positive, while $\mathrm {Age}_{\le 3}$ is negative and highly significant. Thus, the incidence of stroke declines rapidly with age from birth to the age of four, before increasing moderately afterwards, although the age effect is not significant for ischemic strokes between 4 and 18 years of age.

Sex was not a predictor for childhood stroke; the coefficient on sex is non-significant in all six initial fits (models 1, 2, 4, 5, 7, 8).

#### Mortality: 30-day stroke fatalities per 100,000 PYs

Due to only nine childhood stroke 30-day fatalities (all associated with a stroke diagnosis; see also Fig. 1b), no regression model was fitted to the mortality data. Nevertheless, as an ad hoc analysis, a simple Fisher’s exact test for equality of proportions yielded a $p = 0.015$ for a decline in overall 30-day stroke mortality for 0–18 year olds, from 0.255/100k during 2010–2011 to 0.000/100k during 2014–2015, suggesting a decline in childhood stroke mortality in Norway during recent years.^5^

**Table 2:** Descriptive statistics (aggregated over 2010–2015)

Age	TIA		Ischemic		Haemorrhagic		Undetermined		CVA		PY
	EVS	CF	EVS	CF	EVS	CF	EVS	CF	EVS	CF	
*Childhood stroke (0–18)*											
0–3	7	0	69	0	35	2	1	0	111	2	1483k
4–14	11	0	46	3	39	4	5	0	100	7	4071k
15–18	15	0	24	0	20	0	0	0	59	0	1555k
0–18	33	0	139	3	94	6	6	0	270	9	7108k
/100k PY	0.45	0.00	1.92	0.04	1.28	0.09	0.09	0	3.71	0.13	
CF/EVS		0.00		0.02		0.07		0		0.04	
*Stroke in the young (15–54)*											
15–24	65	0	105	1	87	5	2	0	258	6	3930k
15–24${}^{female}$	29	0	55	0	38	0	1	0	123	0	1913k
15–24$^{mars}$	36	0	50	1	49	5	1	0	135	6	2017k
25–34	163	0	344	2	128	12	19	1	648	15	3956k
25–34$^{female}$	87	0	155	2	55	6	10	0	305	8	1936k
25–34$^{mars}$	76	0	189	0	73	6	9	1	343	7	2021k
35–44	648	1	1183	17	299	39	50	4	2162	57	4333k
35–44$^{female}$	260	0	482	7	114	14	19	0	870	20	2105k
35–44$^{mars}$	388	1	701	10	185	25	31	4	1292	37	2228k
45–54	1936	0	3245	66	628	123	135	23	5905	201	4084k
45–54$^{female}$	735	0	1087	23	223	43	55	5	2084	68	1986k
45–54$^{mars}$	1201	0	2158	43	405	80	80	18	3821	133	2098k
15–54	2812	1	4877	86	1142	179	206	28	8973	279	16,304k
15–54$^{female}$	1111	0	1779	32	430	63	85	5	3382	96	7940k
15–54${}^{mars}$	1701	1	3098	54	712	116	121	23	5591	183	8364k
/100k PY	17.25	0.01	29.91	0.53	7	1.10	1.26	0.17	55.04	1.71	
/100k PY$^{female}$	13.99	0.00	22.41	0.40	5.42	0.79	1.07	0.06	42.59	1.21	
/100k PY${}^{mars}$	20.34	0.01	37.04	0.65	8.51	1.39	1.45	0.27	66.85	2.19	
CF/EVS		0.00		0.02		0.16		0.14		0.03	
CF/EVS$^{female}$		0.00		0.02		0.15		0.06		0.03	
CF/EVS${}^{mars}$		0.00		0.02		0.16		0.19		0.03	

${}^{female}$ Females; ${}^{mars}$ Males; CVA = TIA + Ischemic + Haemorrhagic + Undetermined; PY = (1000) Person years ; EVS = Total cases registered, CF = case fatalities; /100k PY = cases per 100,000 PYs (crude incidence rate, mortality rate), CF/EVS = 30-day case fatality rate

#### 30-day stroke case fatality rate

Overall case fatality rate for the 0–18 group was 3.8%. A similar rudimentary analysis by means of Fisher’s test as for the 30-day overall stroke mortality, comparing 2010–2011 vs. 2014–2015 suggests that the 30-day case fatality rate dropped from 7.06 to 0.00% ($p = 0.029$).^6^

**Table 3:** Regression output from childhood stroke negative binomial regression models

	Ischemic			Haemorrhagic			Strokes		
	Model 1	Model 2	Model 3	Model 4	Model 5	Model 6	Model 7	Model 8	Model 9
$\mathrm {Year}$	0.034	0.063		0.019	0.009		0.021	0.022	
(sd)	(0.078)	(0.070)		(0.075)	(0.069)		(0.057)	(0.048)	
$\mathrm {Age}$	$-\,$0.073**	0.046	0.044	$-\,$0.034	0.066*	0.066*	$-\,$0.057**	0.048*	0.048*
(sd)	(0.025)	(0.033)	(0.033)	(0.023)	(0.033)	(0.034)	(0.018)	(0.023)	(0.023)
$\text {Age}_{\le 3}$		$-\,$1.062***	$-\,$1.051***		$-\,$1.013***	$-\,$1.013***		$-\,$1.034***	$-\,$1.033***
(sd)		(0.226)	(0.226)		(0.241)	(0.241)		(0.161)	(0.161)
$\mathrm {Age}\times \text {Age}_{\le 3}$		2.847***	2.808***		2.528***	2.525***		2.607***	2.605***
(sd)		(0.515)	(0.514)		(0.512)	(0.513)		(0.353)	(0.354)
$\mathrm {Sex}$	$-\,$0.126	$-\,$0.137		$-\,$0.188	$-\,$0.205		$-\,$0.137	$-\,$0.129	
(sd)	(0.268)	(0.240)		(0.256)	(0.238)		(0.195)	(0.166)	
EVS	94	94	94	139	139	139	237	237	237
PY	7108132	7108132	7108132	7108132	7108132	7108132	7108132	7108132	7108132
*n*	228	228	228	228	228	228	228	228	228
LogLik	$-\,$230.558	$-\,$213.728	$-\,$214.343	$-\,$193.924	$-\,$179.981	$-\,$180.368	$-\,$317.537	$-\,$287.728	$-\,$288.163
$\theta$	0.443***	0.908**	0.902**	0.782**	1.859	1.822	0.862***	2.472**	2.437**
(sd)	(0.108)	(0.309)	(0.310)	(0.288)	(1.084)	(1.056)	(0.183)	(0.933)	(0.915)
AIC	469.116	439.456	436.686	395.848	371.961	368.737	643.073	587.456	584.327

Notes: $$***p< .001$$; $${**}p < .01$$; $${*}p < .05$$. EVS = number of cases recorded, PY = person years, *n* = number of observations (i.e., year–age–sex cells), LogLik = log-likelihood, $$\theta$$ = the estimated dispersion parameter, AIC = Akaike Information Criterion, Strokes = Ischemic + Haemorrhagic + Undetermined.

Models 1, 4, 7 are without the $\mathrm {Age}\times \text {Age}_{\le 3}$ interaction; models 3, 6, 9 are without the non-significant year and sex variables. $\theta$ is a parameter quantifying (inverse) over dispersion: a large value indicates a Poisson model is equally well-suited. The intercepts are not shown

### Young stroke ($15\le \mathrm {Age}\le 54$)

#### Incidence: CVAs per 100,000 PYs

The main regression analysis with respect to incidence is presented in Table 4 (models 1, 3, 5), where the independent variable was the CVA-count.

The effects of both age and sex on CVA incidence were strong and highly significant in the model fitted to the whole data set. There is a non-significant ($p = 0.052$) and negative temporal trend for the combined model, and this trend is not significant in the sex-specific models either; the sign of the coefficient on year is negative whenever it is included alone, or very near zero when paired with the interaction.

Similar models were fitted for each of the categories ischemic, haemorrhagic, undetermined, strokes, and TIA, for both sexes, and for females and males separately. Furthermore, these specifications were also fitted to each 10-year strata, to investigate if any sub-strata stood out ominously.

Alongside the coefficient on year and its *p* value, we have reported the estimated difference in expected number of events per 100k PYs for 2010–2011 vs. 2014–2015, and the *p* value for this difference. Crude changes in incidence rates, results from Chi square test and regression modelling of the incidence rates of all six diagnoses fitted to data stratified by 10-year age groups and by sex are reported in Table 5; only the coefficient on year ($\beta$) is reported.

The Mann–Kendall non-parametric test for temporal trend gave no significant results of interest (the monotonic negative trend for undetermined stroke yielded two $p=0.024$) and is not reported.

**Fig. 2:**
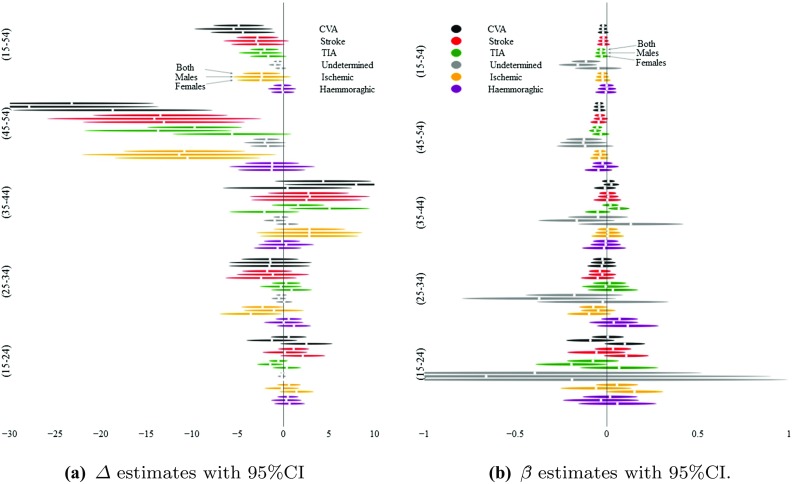
Panel **a** depicts the $\varDelta$ estimates (change in events per 100k PYs between 2010–2011 vs. 2014–2015) with their associated 95% CIs. Similarly, panel **b** depicts ${\hat{\beta }}$ estimates with 95% CIs. In both panels, estimates are grouped by age bracket, sub-diagnosis (colour), and sex, in repeating order. That is, within each main group, estimates for CVAs are given at the top, and estimates for haemorrhagic strokes at the bottom, while within a diagnosis, estimates are given for both sexes above males above females. See also Table 5 for coefficients and *p* values

A majority of the $\beta$-estimates (63/90) and $\varDelta$-estimates (59/90) were negative, although very few were significant; Fig. 2a, b present a graphical impression of the estimates’ uncertainty. For the entire group of 15–54 year olds, there has been a decline of about 3 strokes per 100k PYs over the six-year period under study ($\varDelta$ for 2010–2011 vs. 2014–2015).

This decline was steeper for males than for females. The total number of CVAs decreased by about 5 per 100k PYs in the period, with a somewhat higher incidence rate among males compared to females. The decrease in this age bracket appear to be driven by a decrease in all diagnosis groups except haemorrhagic strokes, and the results of the age-stratified analysis suggest that the decrease is mostly occurring in the 45–54 bracket. It is also here that the majority of CVAs occur: 54.5% of the CVAs in individuals between 15 and 54 occurred in that age bracket. There were overall increase in both the 15–24 and 34–44 age groups, and a decrease in the 25–34 age group, but none of these are statistically significant.

Considering that most of the CVAs occur in the 45–54 group, combined with the observation that this is the only age group that display a significant temporal trend, we support the concern discussed above about how a decline in one part of the data could mask an increase in another part. This suggests^7^ the possibility of including a dummy for age above 44—analogous to the $\mathrm {Age}_{\le 3}$-dummy used in the childhood CVA specification—along with its interaction with year to the regression model specification.

We therefore fitted also this model to the CVA incidence data (and to the stroke-mortality data), the results are presented in Table 4 (even numbered models.) The model largely confirms its underlying hypothesis: the coefficient on year all but vanishes when the model has an extra degree of freedom to estimate a different trend for the sub-group above 45 and those below that age. Instead, we see a weakly ($p=0.028$) significant and negative coefficient on the $\mathrm {Year}\times \mathrm {Age}_{\ge 45}$-interaction’s coefficient, and an AIC that supports the inclusion of these variables.

**Table 4:** Regression output from CVA in the young negative binomial regression models

	CVA incidence						Stroke 30-day overall mortality					
	Both		Females		Males		Both		Females		Males	
	Model 1	Model 2	Model 3	Model 4	Model 5	Model 6	Model 7	Model 8	Model 9	Model 10	Model 11	Model 12
Year	$-\,$0.019	0.001	$-\,$0.019	$-\,$0.0001	$-\,$0.020	0.005	$-\,$0.063	$-\,$0.075	$-\,$0.071	$-\,$0.016	$-\,$0.059	$-\,$0.110
(sd)	(0.010)	(0.013)	(0.015)	(0.020)	(0.012)	(0.017)	(0.035)	(0.067)	(0.060)	(0.111)	(0.044)	(0.085)
$\mathrm {Year}\times \mathrm {Age}_{\ge 45}$		$-\,$0.042*		$-\,$0.040		$-\,$0.047*		0.016		$-\,$0.078		0.068
(sd)		(0.019)		(0.029)		(0.024)		(0.079)		(0.132)		(0.099)
$\mathrm {Age}_{\ge 45}$		85.582*		80.669		94.866*		$-\,$32.673		156.079		$-\,$137.181
(sd)		(38.978)		(59.128)		(47.854)		(158.677)		(265.364)		(198.517)
$\mathrm {Age}$	0.106***	0.104***	0.095***	0.095***	0.115***	0.111***	0.126***	0.115***	0.132***	0.145***	0.123***	0.100***
(sd)	(0.002)	(0.003)	(0.003)	(0.004)	(0.002)	(0.004)	(0.008)	(0.014)	(0.015)	(0.026)	(0.010)	(0.017)
Sex	0.382***	0.383***					0.587***	0.587***				
(sd)	(0.034)	(0.033)					(0.126)	(0.126)				
EVS	8973	8973	3382	3382	5591	5591	278	278	96	96	182	182
PY	16304k	16304k	7940k	7940k	8364k	8364k	16304k	16304k	7940k	7940k	8364k	8364k
*n*	480	480	240	240	240	240	480	480	240	240	240	240
LogLik	$-\,$1346.089	$-\,$1343.234	$-\,$642.513	$-\,$641.592	$-\,$688.023	$-\,$685.212	$-\,$358.344	$-\,$357.887	$-\,$149.075	$-\,$148.709	$-\,$210.115	$-\,$208.616
$\theta$	21.055***	21.964***	19.644***	20.001***	29.693***	32.246***	618.559	958.350	1096.943	1062.434	390.639	1362.031
(sd)	(3.381)	(3.596)	(4.750)	(4.886)	(6.886)	(7.677)	(8232.034)	(11030.020)	(19115.880)	(17751.840)	(7106.713)	(17026.560)
AIC	2700.178	2698.469	1291.026	1293.184	1382.046	1380.423	724.687	727.774	304.151	307.418	426.231	427.231

Notes: $$***\hbox {p} < .001$$; $$**\hbox {p} < .01$$; $${*}\hbox {p} < .05$$ EVS = number of CVA events recorded, PY = person years, *n* = number of observations (i.e. year-age-sex cells), LogLik = log-likelihood, $$\theta$$ = the estimated dispersion parameter, AIC = Akaike Information Criterion, Stroke = Ischemic + Haemorrhagic + Undetermined; CVAs = Stroke + TIA Models 1–6 fitted to CVA incidence data; 1 and 2 include a Sex-dummy. Models 3 and 4 (5 and 6) are fitted for Females (Males) separately. Models 7–12 fitted to Stroke 30-day case fatality data; 7 and 8 include a Sex-dummy. Models 9 and 10 (11 and 12) are fitted for Females (Males) separately. Intercepts are not shown

#### Mortality: 30-day stroke fatalities per 100,000 PYs

There were very few 30-day case-fatalities following TIAs. Therefore, the same main models that were fitted for CVA incidence were fitted only for fatalities following within 30 days of a stroke. The results of the main regressions are presented in Table 4 (models 7,9,11), we see again a non-significant ($p = 0.074$) negative temporal trend towards lower stroke mortality, and this time, the model with the $\mathrm {Age}_{\ge 45}$-terms did not show a better fit, suggesting that mortality—if declining—is *not* specific to the 45–54 age band.

We also note that the $\theta$-estimate is non-significant, suggesting that a simpler Poisson model is more appropriate. When fitted to the data, a marginal increase in AIC was observed, the coefficient on Year remained negative ($-0.063$; $p = 0.073$) yielding an essentially identical model.

For the sub-group analyses, we also only fitted models to the haemorrhagic, ischemic, and stroke mortality data. Similar patterns as for incidences are observed, see Table 6, and again the all non-significant Mann–Kendal results are omitted.

Contrasting the CVA-incidence models, there were few significant results, and very wide CIs for the coefficients on the year variable.^8^ A majority of the $\beta$-estimates (39/45) and $\varDelta$-estimates (32/45) were negative.

**Table 5:** Estimates of crude incidence rates 2010–2015; regression modelling (${\hat{\beta }}$ estimates temporal trends), and comparison of stroke incidence rates in 2010–2011 vs. 2014–2015

Age		Ischemic		Haemorrhagic		Undetermined		Stroke		TIA		CVA	
		Est.	*p* val. or 95% CI	Est.	*p* val. or 95% CI	Est.	*p* val. or 95% CI	Est.	*p* val. or 95% CI	Est.	*p* val. or 95% CI	Est.	*p* val. or 95% CI
15–24													
Male	${\hat{I}}$	2.5 (1.7–3.3)		2.4 (1.7–3.2)		0.0 ($-\,$0.1 to 0.2)		4.9 (3.8–6.0)		1.8 (1.1–2.5)		6.7 (5.5–7.9)	
	${\hat{\beta }}$	$-\,$0.058	0.563	$-\,$0.031	0.769	$-\,$0.661	0.407	$-\,$0.056	0.495	$-\,$0.193	0.055	$-\,$0.089	0.191
	${\hat{\varDelta }}$	$-\,$0.152	0.998	0.335	0.812	$-\,$0.154	0.977	0.183	0.978	$-\,$1.425	0.050	$-\,$1.242	0.419
Female	${\hat{I}}$	2.9 (2.0–3.7)		2.0 (1.3–2.7)		0.1 ($-\,$0.2 to 0.3)		4.9 (3.8–6.0)		1.5 (0.9–2.2)		6.4 (5.2–7.7)	
	${\hat{\beta }}$	0.153	0.060	0.059	0.592	$-\,$0.190	0.753	0.108	0.088	0.069	0.528	0.100	0.079
	${\hat{\varDelta }}$	1.453	0.146	0.692	0.497	0.000	1.000	2.146	0.095	0.392	0.745	2.538	0.087
Both	${\hat{I}}$	2.7 (2.1–3.2)		2.2 (1.7–2.7)		0.1 ($-\,$0.1 to 0.2)		4.9 (4.2–5.7)		1.7 (1.2–2.1)		6.6 (5.7–7.4)	
	${\hat{\beta }}$	0.057	0.334	0.019	0.802	$-\,$0.394	0.400	0.035	0.498	$-\,$0.075	0.308	0.007	0.872
	${\hat{\varDelta }}$	0.630	0.382	0.509	0.435	$-\,$0.079	0.979	1.139	0.206	$-\,$0.541	0.338	0.598	0.591
25–34													
Male	${\hat{I}}$	9.4 (7.9–10.8)		3.6 (2.7–4.5)		0.4 (0.1–0.8)		13.2 (11.5–14.9)		3.8 (2.8–4.7)		17.0 (15.1–18.9)	
	${\hat{\beta }}$	$-\,$0.046	0.344	0.039	0.572	$-\,$0.370	0.087	$-\,$0.024	0.534	$-\,$0.005	0.936	$-\,$0.019	0.573
	${\hat{\varDelta }}$	$-\,$1.021	0.587	0.051	1.000	$-\,$0.483	0.314	$-\,$1.141	0.611	$-\,$0.261	0.918	$-\,$1.403	0.576
Female	${\hat{I}}$	8.0 (6.6–9.4)		2.8 (2.0–3.7)		0.5 (0.1–0.9)		11.3 (9.7–12.9)		4.5 (3.4–5.5)		15.8 (13.9–17.6)	
	${\hat{\beta }}$	$-\,$0.096	0.118	0.116	0.183	$-\,$0.021	0.910	$-\,$0.049	0.288	0.034	0.631	$-\,$0.026	0.526
	${\hat{\varDelta }}$	$-\,$3.561	0.039	1.170	0.253	0.107	1.000	$-\,$2.431	0.234	0.914	0.479	$-\,$1.517	0.535
Both	${\hat{I}}$	8.7 (7.7–9.7)		3.2 (2.6–3.8)		0.5 (0.2–0.7)		12.3 (11.1–13.4)		4.1 (3.4–4.8)		16.4 (15.1–17.7)	
	${\hat{\beta }}$	$-\,$0.073	0.065	0.071	0.172	$-\,$0.177	0.196	$-\,$0.037	0.225	0.016	0.741	$-\,$0.023	0.389
	${\hat{\varDelta }}$	$-\,$2.266	0.057	0.604	0.453	$-\,$0.195	0.651	$-\,$1.770	0.216	0.315	0.755	$-\,$1.455	0.380
35–44													
Male	${\hat{I}}$	31.5 (29.0–33.9)		8.3 (7.0–9.6)		1.4 (0.8–2.0)		40.6 (37.8–43.3)		17.4 (15.6–19.2)		58.0 (54.7–61.2)	
	${\hat{\beta }}$	0.007	0.850	$-\,$0.005	0.912	$-\,$0.164	0.129	0.009	0.762	0.067	0.024	0.026	0.222
	${\hat{\varDelta }}$	2.866	0.349	0.315	0.905	$-\,$0.937	0.149	2.915	0.400	5.083	0.024	7.998	0.047
Female	${\hat{I}}$	22.9 (20.8–25.0)		5.4 (4.3–6.5)		0.9 (0.4–1.4)		29.0 (26.6–31.4)		12.3 (10.8–13.9)		41.3 (38.5–44.2)	
	${\hat{\beta }}$	0.006	0.898	$-\,$0.014	0.819	0.133	0.367	0.004	0.926	$-\,$0.049	0.181	$-\,$0.009	0.776
	${\hat{\varDelta }}$	2.859	0.314	$-\,$0.617	0.708	0.446	0.583	2.542	0.427	$-\,$2.071	0.305	0.470	0.927
Both	${\hat{I}}$	27.3 (25.7–28.9)		6.9 (6.1–7.7)		1.2 (0.8–1.5)		34.9 (33.1–36.7)		15.0 (13.8–16.2)		49.9 (47.7–52.0)	
	${\hat{\beta }}$	0.006	0.825	$-\,$0.005	0.898	$-\,$0.047	0.579	0.006	0.787	0.021	0.371	0.010	0.572
	${\hat{\varDelta }}$	2.880	0.158	$-\,$0.130	0.951	$-\,$0.265	0.618	2.759	0.231	1.623	0.284	4.381	0.107
45–54													
Male	${\hat{I}}$	102.8 (98.4–107.3)		19.3 (17.3–21.3)		3.8 (2.9–4.7)		124.9 (120.0–129.7)		57.2 (53.9–60.6)		182.1 (176.2–187.9)	
	${\hat{\beta }}$	$-\,$0.033	0.127	$-\,$0.007	0.863	$-\,$0.128	0.053	$-\,$0.032	0.105	$-\,$0.063	0.000	$-\,$0.041	0.006
	${\hat{\varDelta }}$	$-\,$11.424	0.036	$-\,$1.223	0.643	$-\,$2.017	0.085	$-\,$14.147	0.018	$-\,$13.731	0.001	$-\,$27.878	0.000
Female	${\hat{I}}$	54.7 (51.4–58.1)		11.2 (9.7–12.8)		2.8 (1.9–3.6)		67.9 (64.2–71.7)		37.0 (34.2–39.8)		104.9 (100.3–109.5)	
	${\hat{\beta }}$	$-\,$0.040	0.126	$-\,$0.045	0.256	$-\,$0.118	0.139	$-\,$0.044	0.055	$-\,$0.032	0.180	$-\,$0.040	0.025
	${\hat{\varDelta }}$	$-\,$10.506	0.010	$-\,$1.270	0.537	$-\,$1.677	0.092	$-\,$13.095	0.004	$-\,$5.615	0.093	$-\,$18.710	0.001
Both	${\hat{I}}$	79.4 (76.7–82.2)		15.4 (14.1–16.6)		3.3 (2.7–3.9)		97.2 (94.1–100.2)		47.4 (45.2–49.6)		144.6 (140.8–148.3)	
	${\hat{\beta }}$	$-\,$0.036	0.032	$-\,$0.022	0.429	$-\,$0.124	0.015	$-\,$0.037	0.014	$-\,$0.051	0.000	$-\,$0.041	0.000
	${\hat{\varDelta }}$	$-\,$10.849	0.001	$-\,$1.224	0.439	$-\,$1.848	0.013	$-\,$13.482	0.000	$-\,$9.721	0.000	$-\,$23.203	0.000
15–54													
Male	${\hat{I}}$	37.0 (35.7–38.4)		8.5 (7.9–9.2)		1.4 (1.2–1.7)		46.5 (45.0–48.0)		20.3 (19.3–21.3)		66.8 (65.1–68.6)	
	${\hat{\beta }}$	$-\,$0.023	0.210	0.002	0.935	$-\,$0.158	0.004	$-\,$0.018	0.235	$-\,$0.032	0.027	$-\,$0.020	0.108
	${\hat{\varDelta }}$	$-\,$2.348	0.153	$-\,$0.136	0.898	$-\,$0.906	0.007	$-\,$2.968	0.106	$-\,$2.481	0.044	$-\,$5.449	0.013
Female	${\hat{I}}$	22.4 (21.3–23.5)		5.4 (4.9–6.0)		1.1 (0.8–1.3)		28.6 (27.4–29.8)		14.0 (13.1–14.8)		42.6 (41.1–44.1)	
	${\hat{\beta }}$	$-\,$0.021	0.341	$-\,$0.004	0.897	$-\,$0.046	0.480	$-\,$0.017	0.370	$-\,$0.027	0.151	$-\,$0.019	0.207
	${\hat{\varDelta }}$	$-\,$2.477	0.063	$-\,$0.044	0.992	$-\,$0.275	0.410	$-\,$2.783	0.063	$-\,$1.645	0.110	$-\,$4.428	0.014
Both	${\hat{I}}$	29.9 (29.1–30.8)		7.0 (6.6–7.4)		1.3 (1.1–1.4)		37.8 (36.8–38.7)		17.2 (16.6–17.9)		55.0 (53.9–56.2)	
	${\hat{\beta }}$	$-\,$0.022	0.126	0.000	1.000	$-\,$0.113	0.006	$-\,$0.017	0.155	$-\,$0.030	0.011	$-\,$0.019	0.052
	${\hat{\varDelta }}$	$-\,$2.375	0.025	$-\,$0.083	0.898	$-\,$0.598	0.008	$-\,$2.833	0.017	$-\,$2.055	0.010	$-\,$4.888	0.001

*Notes:* For each sex and age group, and each stroke category *SX*, the model $SX \sim \beta \mathrm {Year} + \beta _a\mathrm {Age} + \beta _s\mathrm {Sex}$ was fitted as a negative binomial model. ${\hat{I}}$ = estimated crude incidence rates for 2010–2015, with 95% CI; ${\hat{\beta }}$ = coefficient on Year; $\varDelta$ = estimated difference in expected number of cases per 100k PY for 2010–2011 vs. 2014–2015; the associated *p* value comes from a $\chi ^2$-test for equality of proportions. For a graphical display of the $\beta$ and $\varDelta$ estimates with 95% CI’s, see Fig. 2. Stroke = Ischemic + Haemorrhagic + Undetermined; CVA = TIA + Stroke

#### 30-day stroke case-fatality rate

The overall Stroke case-fatality rate was estimated at 4.5%, ranging from 1.8% for ischemic to 15.7% for haemorrhagic strokes. There was no statistical evidence for a decrease in stroke case-fatality rates, although point estimates were negative (not shown). The crude case-fatality rates are shown in Table 2.

### Non-haemorrhagic vs. haemorrhagic strokes

The observed proportion of ICH of all CVAs for each 1-year age group (2010–2015 aggregated) is plotted in Fig. 1c. There is a discernible trend towards a lower fraction of haemorrhagic strokes with increasing age, although the estimates for the youngest are based on very few events.

## Discussion

We find no evidence for a temporal trend of increased stroke incidence in the young, nor for childhood stroke. Rather, we find weak evidence for a decline in CVAs in the young (15–54), although this trend most likely pertains only to the Strokes in the 45–54 stratum (and TIAs), and appears stationary across the 15–44 group (Table 4 [model 2], Table 5 and Fig. 2). Furthermore, we find some evidence for a decrease in both childhood (0–18) and young stroke *mortality*, consistent with the literature, [17] while childhood CVA incidence appears stationary. This is mirrored in the stationary case-fatality rates for young adults, and an indication of a drop in childhood case-fatality rates.

In Norway, ten years into the millennium, all facts considered, stroke incidence and mortality appear to be stable for children and the younger cohorts, while the decline in stroke incidence for the middle aged that has been observed for quite some time has still not stabilised at a new incidence-level [38].

Our findings thus do not support recent publications suggesting an increase in stroke incidence in the young, and our findings contrast the findings reported on in the studies summarised in Table 1.

As for the clinical relevance of these estimates, keeping in mind that when $\beta$ is close to zero, it can approximately be interpreted as a percentage change per year, we see that the main regression model predicts a decline in CVA incidence of about 4% per year, but only in the 45–54 cohort. In Norway, there are about $7.1\times 100\mathrm {k}$ individuals in this age bracket; from which we currently expect $67.3\times 7.1=478$ CVA events in a given year. About 10% of these patients will die without admission, [28] meaning that the yearly absolute reduction is given by $(0.9\times 478\times 0.96^y)\times 0.04$ between years *y* and $y+1$. The model thus predicts that the number of CVA admissions of patients in the bracket 15–54 will drop from the current 430 to 275 by 2025. It is reasonable to assume—given the ageing of the population, combined with the fact that most CVAs originate with the 55+ cohorts –that stroke units will not see a clinically relevant reduction due to *this* decline.

Our results are valid for Norway—indeed we have data on every CVA known to Norwegian health authorities. We also believe that our results likely generalise to the Scandinavian countries, and possibly other northern European countries (e.g. the Netherlands). We do not venture to assume anything beyond this. It follows that we believe that cited results (Table 1) on increasing trends may be spurious, and that continued inquiry is warranted. See also Mallick et al.’s study for a further discussion of spurious temporal trends [39]. Crucially, such studies should include all results, and follow a pre-set (preferably pre-registered—here we are failing ourselves) protocol for which age-brackets to analyse and sub-analyses to undertake.

Admittedly, our data does not contain more than six years, and although we believe that future data in our series will not negate our results, we are hoping to confirm these findings as more data become available from the NPR and NCDR.

The incidence and mortality rates we report are in keeping with previously published studies. As we report on *all* CVAs (excluding sub-arachnoid haemorrhage), and not only hospitalised CVAs, some variation throughout the literature is to be expected. We do not know of any recent studies that are incompatible with our results. E.g. Marini et al. [40] reported incidence rates for first-ever strokes (including sub-arachnoid haemorrhage) – pooled over 29 studies—at 4.2/100k PY for 15–24; 10.7 for 25–34; and 30.7 for 35–44 (males). These are lower than our estimates at, respectively 4.9,  13.2,  and 40.6, but as we include both recurrent strokes and non-hospitalised strokes registered through the NCDR, these numbers square well. The figures for females compare similarly. Putaala et al. report a 95% CI of (8.4–13.0) for ages 15–49 for first-ever ischemic strokes; we calculate^9^ 20.7 for first-ever and recurrent including non-hospitalised cases. Ischemic- and haemorrhagic stroke mortality in children—0.0 and 0.1/100k PY in our study—also compare well with figures of 0.0 and 0.1 from Krishnamurthi et al. [17]. Béjot et al. reports incidences for first-ever strokes by category for 0–55 as 18.1 (ischemic), 2.2 (haemorrhagic) and 0.0 (undetermined); [6] we find 20.7,  5.3, and 0.9—which fits well with the inclusion of recurrent strokes and the NCDR data on non-hospitalised, fatal strokes.

There are several reasons why others may have found rising trends, and which we believe may have been discussed insufficiently. Chief among these reasons is increased stroke awareness; changes in clinical practice, particularly regarding how patients are handled when minor strokes are suspected; and improved diagnostics during the preceding decade. These mechanisms all work towards biasing estimates on trends in an upward direction. Our study is well positioned to untangle some of these effects, but we cannot rule out that our observation of a temporally stationary incidence profile throughout 2010–2015 is really a moderate decrease in stroke incidence. The observed decline in mortality, which includes non-hospitalised CVA events, corroborates this result [41].

Our study shows that children in Norway have never been safer from stroke fatalities than today. Little seem to have changed with regards to incidence and mortality rates since the late 70s. [10, 39] Furthermore, our results suggest that one is never at a lower risk of stroke, ischemic or haemorrhagic, than when roughly four years of age. The observed stationary or declining incidence in stroke incidence comes despite it being shown that improved imaging techniques may have resulted in earlier estimates being biased downwards [42]. Furthermore, although sex was not a significant predictor in childhood stroke, the Sex-coefficients are all negative (indicating lower incidence for females). There is good reason to believe that male sex is a risk-factor for childhood stroke [10].

Regarding childhood stroke, we see that haemorrhagic strokes are relatively more common in children than adults, as expected. Our results replicate the approximate 50%–50% distribution between ischemic- and haemorrhagic reported for childhood stroke [43]. The incidence rates we find for all CVA in children (3.83/100kPY), and the 30-day case fatality rate (0.13/100kPY) suggest that mortality rates have continued the rather steep decline between 1950 and 2000 in the UK reported on by Mallick et al.; they found a drop from about 0.7/100kPY to about 0.5/100kPY [10]. Broderick et al. and Schoenberg et al. reported CVA 30-day case fatality of about 0.5[43, 44].

We have not paid much attention to discussing the $\varDelta$-estimates (Tables 5 and 6). Although we find slightly different results by this method, than with the regression modelling, we mainly included this analysis to counter possible critiques for not comparing the sub-periods’ point estimates, since this method has been employed in other studies.

The main strength of our study is that we observe the entire population of Norway, meaning that there is no selection bias, other than what may be represented by unequal access to health care. It is conceivable that particularly marginalised groups, e.g. refugees or illegal immigrants, would not be covered by the statistics that underlie our analyses. However, Norway provides free, universal emergency care to all individuals on Norwegian territory, and the fact that our data includes data from the NCDR, means that only very few CVA events would be missed (however, see also below how this is a possible *limitation* of the study.) Access to all data from the entire country also minimises other socio-economic selection mechanisms, which are known to bias results from studies relying on data from private-only, public-only, or regionally specific stroke-centres. In addition to observing (nearly) all strokes, we also have precise information regarding the population at risk, stratified by age and sex; i.e. we know both how many 37-year old women suffered a stroke in 2011, and how many did not. In summary, we believe there are very little missing data, and that any missing data likely pertains to the upper cohorts of the NPR and NCDR data sets, since all suspected CVAs should be seen by a hospital.

A further strength is the use of data that has not been stratified prior to analysis. In our regression models, age has been entered as a continuous variable. This precludes any bias introduced by aggregating by year, in the presence of shifts of the within bracket distribution. As discussed briefly above, aging of the younger combined with the exponential increase in stroke risk that age confers (from the age of four and onwards; recall Fig. 1a), means that any aggregating across age-brackets is susceptible to introduce bias in a temporal trend due to auto-correlation of time and within-bracket age-shift.

Also, it is a strength of this study that we performed a pre-defined battery of analyses (regression modelling, $\chi ^2$, Mann–Kendall), and report on all outcomes. Depending on our own biases, we could have reported an increase in TIA in males aged 35–44 ($p=0.024$), or a decrease in TIA in males aged 15–24 ($p=0.050$). However, when these ‘results’ are shown in Table 5 alongside the other values, it becomes apparent that these are part of a general body of evidence for a stationary, or possibly declining, temporal trend.

The main limitations to our study, despite covering all of Norway, is the sample size; the sub-analyses on 10-year age-groups each contain roughly four million PYs and 50–500 stroke events. Hence, the study could be underpowered to detect an actual temporal trend specific to some of the younger age-brackets investigated. Any such temporal trend, however, cannot be pronounced. Furthermore, our data is limited to the relatively short time frame of 2010–2015.

With respect to 30-day stroke-mortality, however, we maintain that there is weak evidence of a slight decline, and fairly strong evidence against an increase. Furthermore, the second regression model, explicitly including the $\mathrm {Age}_\ge 45$-dummy, provided a point estimate of 0.001 for the overall temporal trend (95% CI − 0.025 to 0.027); which is certainly compatible with a stationary temporal trend.

Another limitation is that the data, in particular data from the NCDR, may be inaccurate. By this we mean that death certificates may be written by physicians without personal knowledge about the anamnesis of the deceased.

Furthermore, we do not have stroke sub-type (e.g. TOAST classification), nor do we distinguish between recurrent and first-ever strokes. On the other hand, we do include TIAs, which we believe is important to get the full picture on CVA trends, and to our knowledge this is the first study to present as detailed numbers on TIA incidence in children and the young as we do. Nevertheless, we believe our results comply with Feigin et al.’s recommendations where the data allows, [30] and it is a further strength of our study that we juxtapose incidences for ischemic, haemorrhagic, and TIA for ready comparison.

**Table 6:** Estimates of crude fatality rates 2010–2015, regression modelling (${\hat{\beta }}$ estimates temporal trends), and comparison of stroke fatality rates in 2010–2011 vs. 2014–2015

Age		Ischemic		Haemorrhagic		Stroke	
		Est.	*p* val. or 95% CI	Est.	*p* val. or 95% CI	Est.	*p* val. or 95% CI
15–24							
Male	${\hat{I}}$	0.0 ($-\,\,$0.1 to 0.2)		0.2 ($-\,\,$0.1 to 0.6)		0.3 (0.0–0.6)	
	${\hat{\beta }}$	$-\,$0.661	0.407	0.074	0.780	$-\,$0.025	0.923
	${\hat{\varDelta }}$	$-\,$0.154	0.977	0.137	1.000	$-\,$0.017	1.000
Female	${\hat{I}}$	0.0 ($-\,$0.1–0.1)		0.0 ($-\,$0.1–0.1)		0.0 ($-\,$0.1–0.1)	
	${\hat{\beta }}$	$-\,$0.012	1.000	$-\,$0.012	1.000	$-\,$0.012	1.000
	${\hat{\varDelta }}$	0.000	1.000	0.000	1.000	0.000	1.000
Both	${\hat{I}}$	0.0 ($-\,$0.1–0.1)		0.1 (0.0–0.3)		0.2 (0.0–0.3)	
	${\hat{\beta }}$	$-\,$0.661	0.407	0.074	0.780	$-\,$0.025	0.923
	${\hat{\varDelta }}$	$-\,$0.079	0.979	0.071	1.000	$-\,$0.008	1.000
25–34							
Male	${\hat{I}}$	0.0 ($-\,$0.1–0.1)		0.3 (0.0–0.6)		0.3 (0.0–0.7)	
	${\hat{\beta }}$	$-\,$0.025	1.000	$-\,$0.022	0.925	$-\,$0.147	0.513
	${\hat{\varDelta }}$	0.000	1.000	$-\,$0.186	0.911	$-\,$0.342	0.595
Female	${\hat{I}}$	0.1 ($-\,$0.1–0.3)		0.3 (0.0–0.7)		0.4 (0.0–0.8)	
	${\hat{\beta }}$	0.162	0.702	$-\,$0.136	0.573	$-\,$0.062	0.766
	${\hat{\varDelta }}$	$-\,$0.014	1.000	$-\,$0.175	0.940	$-\,$0.189	0.922
Both	${\hat{I}}$	0.1 (− 0.1 to 0.2)		0.3 (0.1–0.5)		0.4 (0.1–0.6)	
	${\hat{\beta }}$	0.162	0.702	$-\,$0.079	0.642	$-\,$0.101	0.506
	${\hat{\varDelta }}$	$-\,$0.007	1.000	$-\,$0.180	0.626	$-\,$0.267	0.447
35–44							
Male	${\hat{I}}$	0.4 (0.1–0.8)		1.1 (0.6–1.7)		1.6 (1.0–2.2)	
	${\hat{\beta }}$	$-\,$0.290	0.147	$-\,$0.048	0.721	$-\,$0.111	0.329
	${\hat{\varDelta }}$	$-\,$0.536	0.292	0.007	1.000	$-\,$0.530	0.566
Female	${\hat{I}}$	0.3 (0.0–0.7)		0.7 (0.2–1.1)		0.9 (0.4–1.5)	
	${\hat{\beta }}$	$-\,$0.017	0.942	0.048	0.762	0.030	0.849
	${\hat{\varDelta }}$	0.005	1.000	0.293	0.668	0.154	0.980
Both	${\hat{I}}$	0.4 (0.2–0.6)		0.9 (0.6–1.2)		1.3 (0.9–1.7)	
	${\hat{\beta }}$	$-\,$0.176	0.241	$-\,$0.003	0.975	$-\,$0.057	0.539
	${\hat{\varDelta }}$	$-\,$0.273	0.397	0.148	0.817	$-\,$0.195	0.760
45–54							
Male	${\hat{I}}$	2.0 (1.3–2.8)		3.8 (2.9–4.7)		6.3 (5.2–7.5)	
	${\hat{\beta }}$	$-\,$0.060	0.501	$-\,$0.099	0.134	$-\,$0.041	0.419
	${\hat{\varDelta }}$	$-\,$0.293	0.846	$-\,$1.444	0.232	$-\,$0.909	0.579
Female	${\hat{I}}$	1.2 (0.6–1.7)		2.2 (1.4–2.9)		3.4 (2.5–4.3)	
	${\hat{\beta }}$	$-\,$0.023	0.848	$-\,$0.103	0.253	$-\,$0.093	0.191
	${\hat{\varDelta }}$	0.082	1.000	$-\,$0.596	0.596	$-\,$0.826	0.519
Both	${\hat{I}}$	1.6 (1.2–2.1)		3.0 (2.4–3.6)		4.9 (4.2–5.7)	
	${\hat{\beta }}$	$-\,$0.047	0.513	$-\,$0.100	0.059	$-\,$0.059	0.155
	${\hat{\varDelta }}$	$-\,$0.108	0.944	$-\,$1.027	0.169	$-\,$0.860	0.367
15–54							
Male	${\hat{I}}$	0.6 (0.4–0.8)		1.4 (1.1–1.7)		2.2 (1.8–2.5)	
	${\hat{\beta }}$	$-\,$0.109	0.174	$-\,$0.075	0.167	$-\,$0.059	0.178
	${\hat{\varDelta }}$	$-\,$0.253	0.327	$-\,$0.370	0.311	$-\,$0.450	0.311
Female	${\hat{I}}$	0.4 (0.2–0.6)		0.8 (0.6–1.0)		1.2 (0.9–1.5)	
	${\hat{\beta }}$	$-\,$0.015	0.892	$-\,$0.073	0.323	$-\,$0.071	0.235
	${\hat{\varDelta }}$	0.020	1.000	$-\,$0.109	0.766	$-\,$0.203	0.580
Both	${\hat{I}}$	0.5 (0.4–0.7)		1.1 (0.9–1.3)		1.7 (1.5–1.9)	
	${\hat{\beta }}$	$-\,$0.073	0.255	$-\,$0.075	0.089	$-\,$0.063	0.074
	${\hat{\varDelta }}$	$-\,$0.119	0.477	$-\,$0.241	0.279	$-\,$0.326	0.226

*Notes:* For each sex and age group, and each stroke category *SX*, the model $SX \sim \beta \mathrm {Year} + \beta _a\mathrm {Age} + \beta _s\mathrm {Sex}$ was fitted as a negative binomial model. ${\hat{I}}$ = estimated crude incidence rates for 2010–2015, with 95% CI; ${\hat{\beta }}$ = coefficient on Year; $\varDelta$ = estimated difference in expected number of cases for 2010–2011 vs. 2014–2015; the associated *p* value comes from a $\chi ^2$-test for equality of proportions. Stroke = Ischemic + Haemmoraghic + Undetermined. (CVA/TIA not analysed as TIAs do not result in fatalities per definition.)

## Conclusion

Norwegian CVA incidence rates appear stationary, with a weakly significant decline for the 45–54 age group. Stroke mortality appears to remain stable or declining, for both sexes, and for all age categories. Case-fatality rates are declining in children, and stationary for the young. These results contrast recent results that have raised a concern about a possible increasing trend in the incidence for the young, while confirming the stationary or declining mortality. From a Norwegian perspective, therefore, it seems there is little reason to fear that the young are experiencing increased exposure to stroke risk.

An important caveat is that although we have access to all CVA-events—hospitalised or leading to death—based on more than 21M PYs, our study is not powered to detect slight temporal trends. Therefore, the continued research into this question is warranted, and, wherever possible, data sets pooled over several countries should be employed. Such research should preferably be pre-registered, and should report on all sub-analyses, and should report on the recommended mid-decade age bands.

## Footnotes

1 Please note that since diagnostic data is limited to the assigned diagnostic codes, first-ever strokes are not uniquely identifiable.

2 G45.4 Transient global amnesia is excluded.

3 Some studies have included also 55–64, but these are rather few. Also, choosing 15–54 also lends itself well to further subdivision into, e.g., 15–34 vs. 35–54, or 10- or 5-year intervals.

4 https://www.ssb.no/en/befolkning.

5 Alternatively; from 0.226/100k to 0.028/100k for 2010–2012 vs. 2013–2015 ($p = 0.021$).

6 Alternatively; from 6.96 to 0.82% for 2010–2012 vs. 2013–2015 ($p = 0.036$).

7 This was pointed out by an anonymous referee on an earlier version of this manuscript.

8 As can be seen by considering the estimates’ magnitudes relative to their standard errors.

9 The estimates in this paragraph were computed ad hoc for comparability of age-brackets, and are not reported in the results section.
